# Supplementary material for: Paclitaxel as HIPEC-Drug after Surgical Cytoreduction for Ovarian Peritoneal Metastases: A Randomized Phase III Clinical Trial (HIPECOVA)
Source: Curr Oncol. 2024 Jan 24;31(2):660–71. doi: 10.3390/curroncol31020048 (PMC10888026; doi:10.3390/curroncol31020048)
Supplement: Supplementary file 1 [file curroncol-31-00048-s001.zip › curroncol-2741636-supplementary.pdf]

Supplementary Materials

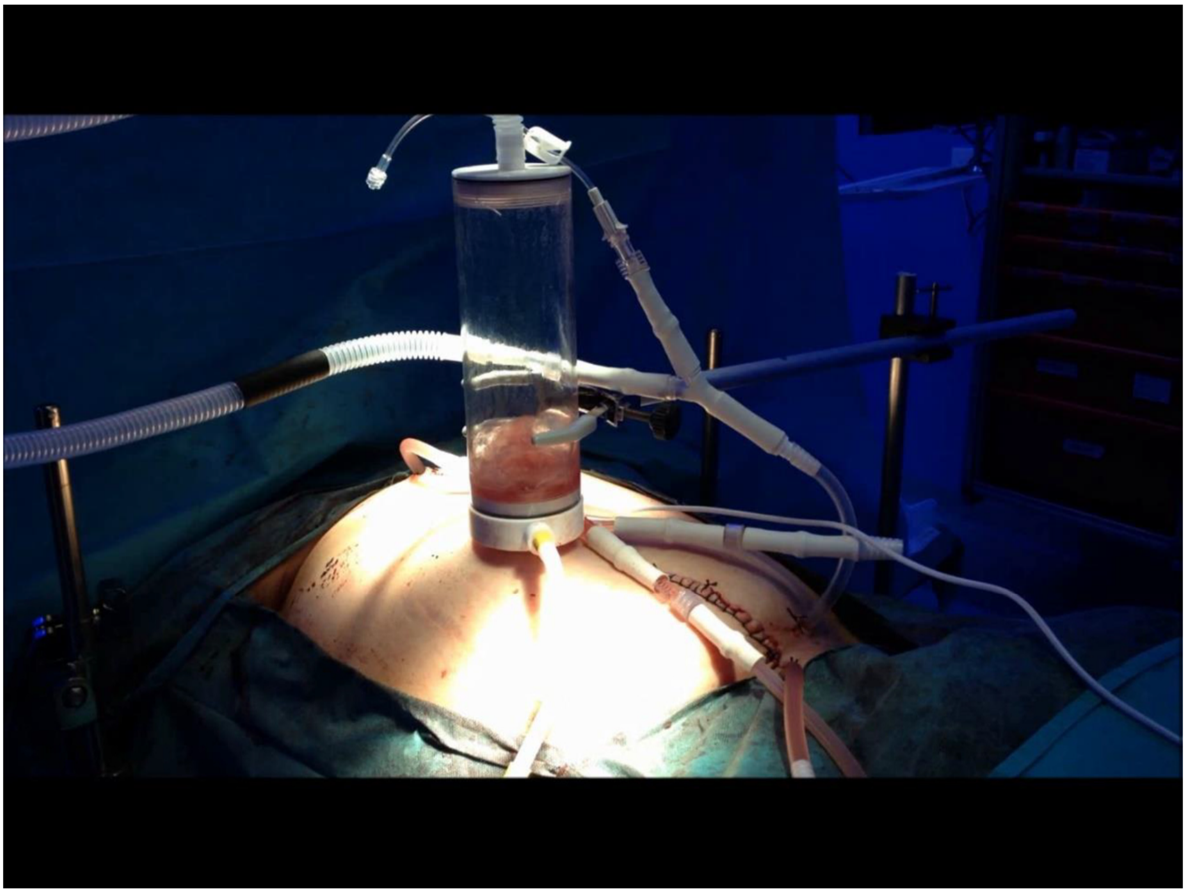

Figure S1. Closed abdomen and CO_2 recirculation system.

Table S1. Peritoneal Surface Disease Severity Score (PSDSS).

Clinical symptoms	Peritoneal cáncer index (PCI)	Tumor histopathology
No symptoms = 0 point	PCI < 10 = 1 point	Well or moderately differentiated and N0 = 1 point
Mild symptoms =1 point	PCI 10-20 = 3 point	Moderately differentiated and N1/N2 = 3 points
Severe symptoms =6 points	PCI > 20 = 7 point	Poorly differentiated or signet ring cell = 9 points

- **Mild symptoms**: weight loss < 10%, mild abdominal pain, asymptomatic ascites.
- **Severe symptoms**: weight loss ≥ 10%, bowel obstruction, uncontrollable pain, symptomatic ascites.

PSDSS Classification	PDSS value
1	2-3 points
2	4-7 points
3	8-10 points
4	>10 points

Table S2. Results of the survival analysis of the main variables related to surgery outcomes.

Variable	OS (median)	Log Rank test	RFS (median)	Log Rank test
**HIPEC**	46	*p* = 0.579	23	*p* = 0.220
**No HIPEC**	48		19	
**Positive lymph nodes**	46	*p* = 0.601	13	*p* = 0.121
**Negative lymph nodes**	51		24	
**Primary cytoreduction**	46	*p* = 0.246	29	*p* = 0.234
**Interval cytoreduction**	36	*p* = 0.584	19	*p* = 0.197
**Secondary cytoreduction**	51	*p* = 0.506	8	*p* = 0.78
**CCR-0 (PCI ≤ 20)**	51	*p* = 0.065	23	*p* = 0.198
**CCR-1 (PCI > 20)**	35		11	
**Intraoperative blood transfusion**	36	*p* = 0.178	13	*p* = 0.47
**No intraoperative blood transfusion**	65		30	
**Histologic Grade I/II**	46	*p* = 0.540	29	*p* = 0.199
**Histologic Grade III**	35	*p* = 0.201	19	

OS: Overall survival, RFS: Recurrence free survival, CCR: Complete Cytoreduction Reduction score, PCI: Peritoneal Cancer Index.

Table S3. Complications according to the Clavien–Dindo Classification.

	**NO adverse events**	**I**	**II**	**IIIa**	**IIIb**	**V**	**TOTAL PATIENTS**
HIPEC	16 (29.1%)	5 (9.1%)	3 (5.5%)	3 (5.5%)	3 (5.5%)	2 (3.6%)	32
NO HIPEC	6 (10.9%)	8 (14.5%)	6 (10.9%)	1 (1.8%)	1 (1.8%)	1 (1.8%)	23
TOTAL	22 (40%)	13 (23.6%)	9 (16.4%)	4 (7.3%)	4 (7.3%)	3 (5.5%)	55

No significant differences between the two groups were noted in adverse event of any grade ($p = 0.482$).
